# Reliability and validity study of the Chinese version of the Cerebellar Cognitive Affective Syndrome Scale in patients with cerebellar injury

**DOI:** 10.1007/s13760-024-02594-x

**Published:** 2024-07-02

**Authors:** Jing Guo, Yi Zhang, Lu Chen, Chaolan Wang, Xiaofang Yuan, Fan Xie

**Affiliations:** Changzhou First People’s Hospital, No. 185, Juqian Street, Tianning District, Changzhou, Jiangsu China

**Keywords:** Cognition, Cerebellum, Scale, Reliability, Validity

## Abstract

**Purpose:**

To preliminarily investigate the reliability and validity of the Chinese version of the Cerebellar Cognitive Affective Syndrome Scale (CCAS scale) in the cerebellar injury population.

**Methods:**

In this study, 40 patients with cerebellar injury and 39 normal individuals hospitalized in a stroke center were assessed using the Chinese version of the CCAS scale A, MMSE, and PHQ2, and the results were analyzed using content validity, structural validity, internal consistency, inter- rater agreement, and test–retest reliability.

**Results:**

The correlation coefficients of semantic fluency, phonemic fluency, category switching, digit span forward, digit span backward, cube, verbal recall, similarities and Go No-Go subscores in the Chinese version of the CCAS scale A were 0.586–0.831 (P ≤ 0.05) with the total score, but there was no significant correlation between the affect and the total score (P = 0.110). The total cognitive score of the Chinese version of the CCAS scale A was correlated with the (r = 0.807, P ≤ 0.01), and the total score of the Chinese version of the CCAS scale A affect was correlated with the total score of PHQ2 (r = 0.884, P ≤ 0.01). The 2 factors were extracted using principal component analysis, and the cumulative variance contribution rate was 59.633%. The factor loadings of each of the corresponding factors were > 0.5, indicating good structural validity of the Chinese version of the CCAS scale A. Cronbach α = 0.827 indicated good internal consistency, and inter-rater reliability (ICC > 0.95) and test–retest reliability (ICC = 0.717–0.895)indicated that the Chinese version of the CCAS scale A had good inter-rater reliability and test–retest reliability.

**Conclusion:**

The Chinese version of the CCAS scale A has good reliability and validity in the cerebellar injury population and is useful for screening cerebellar cognitive-emotional syndrome.

**Supplementary Information:**

The online version contains supplementary material available at 10.1007/s13760-024-02594-x.

## Research background

The Cerebellar Cognitive Affective Syndrome (CCAS), introduced by Schmahmann and Sherman in 1998, describes a clinical syndrome marked by cognitive and affective disturbances following cerebellar injury. This syndrome is characterized by a complex array of symptoms that intertwine cognitive impairments with emotional disorders. Notably, individuals with CCAS may experience attention deficits, reduced verbal fluency, grammatical inaccuracies, visuospatial disorientation, depression, and behavioral abnormalities. These manifestations highlight the cerebellum's significant role in both cognitive functions and emotional regulation [1–4].

Historically, the Mini-Mental State Examination (MMSE) and the Montreal Cognitive Assessment (MoCA) were widely employed in clinical settings to diagnose cerebellar affective-cognitive syndrome; however, they are now deemed inappropriate for this purpose. In 2018, Schmahmann and Sherman developed the Cerebellar Cognitive-Affective Syndrome Scale (CCAS Scale), which subsequent international research has validated for its high sensitivity and specificity in assessing cerebellar cognitive-emotional syndrome. Since its introduction, the CCAS Scale has been translated into several languages and adapted for a range of cerebellar disorders [5–11], yet no studies have validated a Chinese version to date. Patients with cerebellar cognitive-emotional syndrome exhibit distinct symptoms compared to those with post-stroke cognitive impairment, underscoring the necessity for precise and effective diagnostic tools. These tools are crucial for enhancing patient outcomes through tailored rehabilitation strategies. To address this gap, our study evaluates the reliability and validity of the Cerebellar Cognitive Affective Syndrome (CCAS) in a Chinese population with cerebellar injuries. Our findings aim to establish a theoretical foundation for the scales broader application in clinical practice.

## Methods

Forty patients with cerebellar injuries admitted to the stroke center of Changzhou First People's Hospital from 2022.03 to 2022.11 were included in this study. Inclusion criteria: (1) history of new cerebellar injury; diagnosis of cerebellar injury, including cerebral infarction, by a professional neurologist based on CT and MRI; (2) age > 18 years; 3. ability to cooperate in completing the full cognitive assessment; 4. patients who agreed to participate in this study. Exclusion criteria: (1) patients who could not complete the full cognitive assessment due to visual or auditory impairment; (2) patients who refused to participate in this study by themselves or their family members.

### Assessment tools

We used the Chinese version of the CCAS scale provided by the original authors, which is divided into two versions, A and B. To verify the reliability of the retest, only version A was used in this study in the verbal registration part of version A, there was the word Wang Jianguo, which was renamed after a male celebrity in mainland China, and to avoid influence, Wang Jianguo was amended to Zhang Jianguo after discussion with the original author and agreement.

The Chinese version A of the CCAS has 10 items: semantic fluency, phonemic fluency, category switching, digit span forward, digit span backward, cube, verbal recall, similarities, Go No-Go, and affect. Among them, semantic fluency, phonemic fluency, category switching, digit span forward, digit span backward, cube, verbal recall, similarities and Go No-Go, were used to assess cognitive function, and affect was used to assess emotion. All items with raw scores less than or equal to the threshold were scored as a failure, with a standard score of 1 for failure and 0 for passing the standard score. 120 raw scores and 10 standard scores were given for the Chinese version of the CCAS scale. Total standard score = 1: CCAS may exist, total standard score = 2: CCAS is likely to exist, total standard score ≥ 3: CCAS exists.

The Mini-mental State Examination (MMSE) is a scale developed by Folstein et al. in 1975 to assess cognitive functioning and is now widely used around the world [12]. The assessment includes orientation, attention, computation, and recall verbal ability. 30 points on the MMSE scale are considered cognitively impaired depending on the level of education, with scores of ≤ 19 for illiteracy, ≤ 22 for elementary school, and ≤ 26 for secondary school and above. It takes approximately 5–10 min to complete the assessment. The PHQ2 depression screening scale (two-item Patient Health Questionnaire, PHQ-2) is a widely used simple mood screening scale [13, 14]. The PHQ2 has 2 items, each of which is rated 0–3, with a total score of 0–6. A total score of ≥ 3 is considered a mood disorder.

### Process

Three trained rehabilitation physicians performed cognitive assessments on the enrolled patients. The first assessment was conducted by the primary examiner who asked questions and the three assessors scored simultaneously and independently. A second assessment was conducted within 24–48 h after the first assessment, and the second assessment was scored by the primary examiner alone. The first assessment included the Chinese version of the CCAS scale A, MMSE, and PHQ2, and the second assessment included only the Chinese version of the CCAS scale A. At the same time, the primary examiner performed cognitive assessments on 39 healthy adults, including the Chinese version of the CCAS scales A and MMSE.

### Data analysis

SPSS 26.0 statistical software was used for data analysis. We performed an inter- rater reliability analysis for the data of the first assessment, and we performed a test–retest reliability analysis for the data of the second assessment. Data from the Chinese version A of the patient's CCAS scale was analyzed for validity with the MMSE and PHQ2. Internal consistency was expressed by Cronbach's alpha coefficient, and inter-rater reliability was expressed by the intra-group correlation coefficient (ICC). Correlation between the Chinese version of the CCAS scale A and MMSE and PHQ2 scores was expressed by Spearman's correlation coefficient, and P < 0.05 was considered a statistically significant difference. ROC analysis was performed on the data to calculate area under the curve, Jordon's index, sensitivity, specificity.

## Study results

There were 40 patients, 26 males, and 14 females. The average age was 64.88 ±10.72 years. The mean years of education were 7.83 ± 3.84 years, of which 4 cases were illiterate, 14 cases had less than 9 years of education, and 22 cases had 9 years or more of education.

### General information

Table 1 shows the situation of the Chinese version A scores of the CCAS scale, with semantic fluency mean score of 11.05 ± 4.862, phonemic fluency mean score of 3.30±2.875, category switching mean score of 2.58 ± 2.062, digit span forward mean score 5.83 ± 1.738, digit span backward mean score 2.78 ± 1.165, cube mean score 7.95 ± 4.039, and verbal recall mean score 6.98 ± 4.638, similarities mean score 3.85 ± 3.199, Go No-Go mean score 0.65 ± 0.893, affect 4.50 ± 1.617, and total score mean score 49.35 ± 19.57.

**Table 1:** Basic A scores of the Chinese version of the CCAS scale

Projects	Mean ± SD	Highest score
Semantic fluency	11.05 ± 4.862	26
Phonemic fluency	3.30 ± 2.875	19
Category switching	2.58 ± 2.062	15
Digit span forward	5.83 ± 1.738	8
Digit span backward	2.78 ± 1.165	6
Cube	7.95 ± 4.039	15
Verbal recall	6.98 ± 4.638	15
Similarities	3.85 ± 3.199	8
Go No-Go	0.65 ± 0.893	2
Affect	4.50 ± 1.617	6
Total score	49.35 ± 19.57	120

### Reliability analysis

#### Internal consistency reliability

The results showed that the Cronbach coefficient of the Chinese version of the CCAS scale was 0.827, which indicates that the scale has good internal consistency.

#### Inter-assessor consistency

The results in Table 2 show that the ICC of the Chinese version of the CCAS scale A total score was 0.999 (95% CI 0.998, 0.999), the ICC of semantic fluency was 0.997 (95% CI 0.995,0.998), phonemic fluency 0.997 (95% CI 0.995, 0.998), category switching 0.998 (95% CI 0.997, 0.999), digit span forward 0.983 (95% CI 0.970, 0.990), digit span backward0.992 (95% CI 0.986, 0.995), cube 0.999 (95% CI 0.999, 1.000), verbal recall 1.000 (95% CI 1.000, 1.000), similarities 0.992 (95% CI 0.986, 0.995), Go No-Go 0.996 (95% CI 0.994, 0.998), and affect 0.992 (95% CI 0.986, 0.995), which indicates good inter-rater agreement for this scale.

**Table 2:** ICC analysis of the Chinese version of the CCAS scale between assessors for sub-scale A and total score

Projects	ICC	95% CI	P
Semantic fluency	0.997	(0.995, 0.998)	0.000
Phonemic fluency	0.997	(0.995, 0.998)	0.000
Category switching	0.998	(0.997, 0.999)	0.000
Digit span forward	0.983	(0.970, 0.990)	0.000
Digit span backward	0.992	(0.986, 0.995)	0.000
Cube	0.999	(0.999, 1.000)	0.000
Verbal recall	1.000	(1.000, 1.000)	0.000
Similarities	0.992	(0.986, 0.995)	0.000
Go No-Go	0.996	(0.994, 0.998)	0.000
Affect	0.992	(0.986, 0.995)	0.000
Total score	0.999	(0.998, 0.999)	0.000

#### Test–retest reliability

Table 3 shows that the retest ICC coefficient of the Chinese version of the CCAS scale A total score was 0.836, and the retest ICC coefficients of semantic fluency, phonemic fluency, category switching, digit span forward, digit span backward, cube, verbal recall, similarities, Go No-Go, and affect were 0.717–0.895, which indicates that the scale has good test–retest reliability.

**Table 3:** Retest reliability of the Chinese version of the CCAS scale for sub-item A and total score

Projects	ICC	r	P
Semantic fluency	0.773	0.635	0.000
Phonemic fluency	0.895	0.823	0.000
Category switching	0.733	0.576	0.000
Digit span forward	0.778	0.640	0.000
Digit span backward	0.722	0.578	0.000
Cube	0.717	0.596	0.000
Verbal recall	0.803	0.690	0.000
Similarities	0.836	0.713	0.000
Go No-Go	0.744	0.587	0.000
Affect	0.846	0.730	0.000
Total score	0.836	0.727	0.000

### Validity

#### Content validity

Table 4 shows the correlation between the sub-scores of the Chinese version of the CCAS scale A and the total score. semantic fluency, phonemic fluency, category switching, digit span forward, digit span backward, cube, verbal recall, similarities and Go No-Go showed a moderate positive correlation with the total score with correlation coefficients of 0.586–0.831. However, there was no significant correlation between the affect and the total score (P = 0.110).

**Table 4:** Correlation between sub-scores and total scores of Chinese version A of the CCAS scale

Projects	r	P
Semantic fluency	0.727	0.000
Phonemic fluency	0.719	0.000
Category switching	0.615	0.000
Digit span forward	0.610	0.000
Digit span backward	0.586	0.000
Cube	0.784	0.000
Verbal recall	0.831	0.000
Similarities	0.684	0.000
Go No-Go	0.629	0.000
Affect	0.257	0.110

#### Principal component factor analysis

Tables 5 and 6 show the principal component factor analysis performed on the sub-scales to clarify the structural validity of the scale. The Kaiser–Meyer–Olkim (KMO) test and Bartlett test were used to determine the feasibility of the factor analysis. the KMO value was 0.754 and the Bartlett sphericity test value was 163.343 with df = 45, P < 0.00, indicating that the factor analysis was appropriate. Using principal component analysis and orthogonal rotation methods, a total of two initial factors were obtained with a cumulative variance contribution of 59.633%.

**Table 5:** Common factor variance

Projects	Initial	Extraction
Semantic fluency	1.000	0.590
Phonemic fluency	1.000 1.000	0.471 0.792
Category switching	1.000	0.384
Digit span forward	1.000	0.549
Digit span backward	1.000	0.624
Cube	1.000	0.698
Verbal recall	1.000 1.000	0.631 0.517
Similarities	1.000	0.706

**Table 6:** Variance and cumulative contribution margin

Ingredients	Initial Eigenvalue			Extraction of the sum of squares of loads			Sum of rotation load squares		
	Total	Variance %	Cumulative %	Total	Variance %	Cumulative %	Total	Variance %	Cumulative %
1	4.546	45.464	45.464	4.546	45.464	45.464	3.17	31.770	31.770
2	1.417	14.168	59.633	1.417	14.168	59.633	2.786	27.862	59.633

Table 7 shows that factor 1 was associated with semantic fluency, category switching, digit span backward, and verbal recall, and cue factor 1 was associated with language, attention, and memory. Factor 2 was associated with Phonemic fluency, cube, verbal recall, similarities, Go No-Go, and affect, and cue factor 2 was associated with language, visual space, executive function, memory, and emotion.

**Table 7:** Rotated component matrix

Factor	1	2
Semantic fluency	0.740	
Phonemic fluency		0.584
Category switching	0.890	
Digit span forward		
Digit span backward	0.674	
Cube		0.647
Verbal recall	0.655	0.518
Similarities		0.688
Go No-Go		0.674
Affect		0.736

#### Convergent validity

Table 8 shows that the MMSE with high reliability and validity was used as the validity standard for the cognitive total score (assessment items other than emotion) of the CCAS scale, and the results showed that the MMSE total score was 23.4 ± 5.063, and the Chinese version of the CCAS scale had a high positive correlation between the A cognitive total score and the MMSE total score (r = 0.818, P ≤ 0.01). PHQ2 was used as the total effective score validity scale in this study, and the results showed that the mean value of PHQ2: was 1.48 ± 1.894, the total affective score of 4.50 ± 1.617, and the total effective score showed a high positive correlation with PHQ2 (r = 0.891, P ≤ 0.01).

**Table 8:** Correlation between CCAS Chinese version A score and MMSE total score, PHQ2

	Mean ± SD	r	P
MMSE	23.4 ± 5.063	0.818	0.000
A total cognitive score of CCAS	44.85 ± 18.712		
PHQ2	1.48 ± 1.894	0.891	0.000
Affect	4.50 ± 1.617		

### ROC curve analysis

In this study, the ROC curve analysis was performed on the total score of the Chinese version of the CCAS, and the results showed that the area under the curve was 0.882, and the Yoden index was calculated according to the formula, and the optimal cutoff value for the total score of the Chinese version of the CCAS was 67.5 (Yoden index was 0.66, sensitivity was 81%, and specificity was 85%).
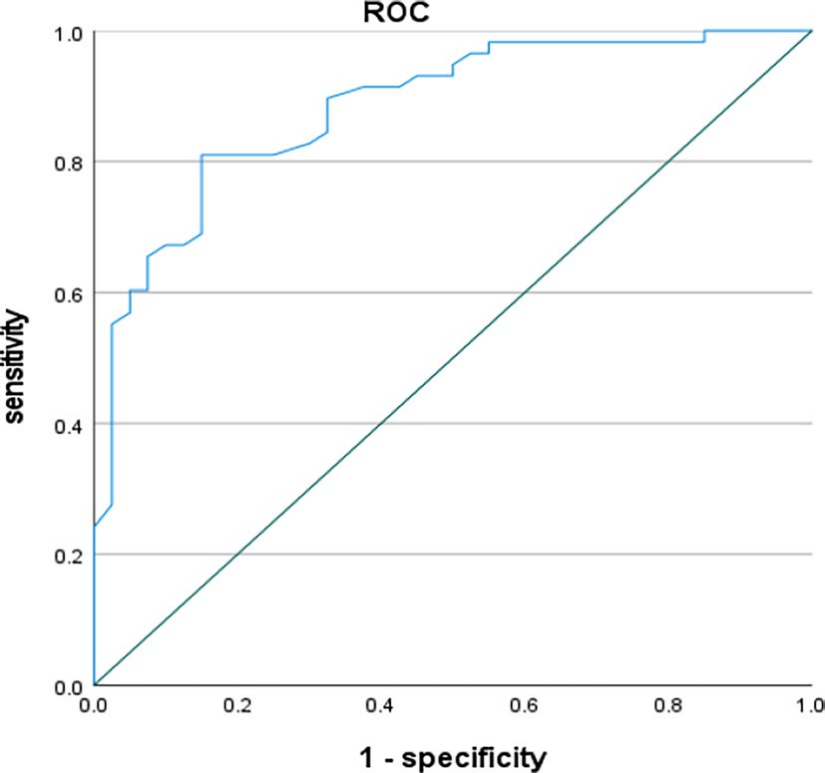


## Discussion

The impairments in executive function, speech function, spatial cognition, and emotions manifested by cerebellar cognitive- emotional syndrome severely affect the ability to perform daily activities and quality of life in patients with cerebellar injury. The cause of cognitive-emotional impairment in patients with cerebellar injury may be the disruption of cerebellar and limbic, prefrontal, and parietal cortical pathways [15, 16].

Currently, for cognitive assessment of cerebellar injury patients, cognitive scales such as MMSE and MoCA and emotional scales such as PHQ2 and PHQ9 are commonly used clinically, but the existing cognitive scales do not reflect emotional disorders, and there are already emotional scales that do not reflect cognitive disorders, so the CCAS scale is a combination of cognitive and emotional assessment and is more suitable for clinical screening of cerebellar cognitive-emotional disorder syndrome. The Chinese version of CCAS Scale A has a total score of 120, which covers verbal function (37.5%), executive function (25.8%), visuospatial function (12.5%), memory function (12.5%), abstract reasoning (6.7%), and emotion (5%). Since the CCAS scale is a new scale, only the English and German versions have completed reliability studies, but all have shown good reliability [5, 8].

In this study, only raw scores were used for analysis, and validity analysis showed that the correlation coefficient between the Chinese version of the CCAS scale A cognitive item score and the total score was between 0.586 and 0.831, which was moderate to highly significant, indicating that the scale had good content validity. Meanwhile, the total cognitive score of the Chinese version of the CCAS scale A was highly correlated with the total score of MMSE (r = 0.818, P ≤ 0.01), and the emotional item score of the Chinese version of CCAS scale A was highly correlated with the total score of PHQ2 (r = 0.891, P ≤ 0.01) indicating that the Chinese version of CCAS scale A has good validity. In addition, factor analysis was used to examine the structural validity of the scale. A total of 2 common factors were extracted, the cumulative variance contribution rate was 59.633%, and the factor loadings of each item were > 0.50, indicating that the Chinese version of CCAS Scale A has good structural validity.

The above findings indicate that the Chinese version A of the CCAS scale has high validity when applied to the Chinese population.

From the reliability analysis, this study used the most commonly used reliability coefficient, Cronbach' s alpha coefficient, which was 0.827, greater than 0.7, indicating that the Chinese version of the CCAS scale A has good internal consistency. The inter-rater agreement reliability was high, with the ICC of the item scores and the total score ranging from 0.982 to 1.000, indicating a high inter-operator agreement. the test–retest reliability of the total score of the Chinese version of the CCAS scale A was 0.836, and the test–retest reliability of the item scores also ranged from 0.717 to 0.895, indicating that the Chinese version of the CCAS scale A has good inter-temporal stability. These indicate that the Chinese version of the CCAS scale A has good reliability in assessing cerebellar cognitive-emotional syndrome.

## Conclusion

The Chinese version of the CCAS Scale A has demonstrated good reliability and validity within the Chinese population affected by cerebellar injuries. This suggests that the scale is well-suited for clinical implementation to deliver targeted cognitive training. Such interventions aim to enhance cognitive functions and improve the daily living activities of patients, potentially leading to better overall rehabilitation outcomes [6, 17]^.^

The limitations of this study are the older age of the subjects and the lower years of education, the difficulty of the Chinese version of the CCAS scale A is difficult for the subjects, and the original authors also suggest that the CCAS scale is recommended for patients under 50 years of age with a higher level of education [18, 19]. The original authors also suggested that the Chinese version of the CCAS scale is recommended for patients under 50 years of age with a high level of education, and further research on the Chinese version of the CCAS scale can be conducted for age and education. The present study only analyzed the raw scores, and further analysis of the Chinese version of the CCAS scale can be conducted to determine the appropriate sub-scale boundaries for the Chinese version.

## Supplementary Information

Below is the link to the electronic supplementary material.

Supplementary file1 (PDF 293 KB)

Supplementary file2 (PDF 257 KB)

## Data Availability

The authors confirm that the data supporting the findings of this study are available within the article and its supplementary materials.
