# Supplementary material for: Reliability and validity study of the Chinese version of the Cerebellar Cognitive Affective Syndrome Scale in patients with cerebellar injury
Source: Acta Neurol Belg. 2024 Jul 2;124(6):1867–73. doi: 10.1007/s13760-024-02594-x (PMC11615023; doi:10.1007/s13760-024-02594-x)
Supplement: Supplementary file 1 — Supplementary file1 (PDF 293 KB) [file 13760_2024_2594_MOESM1_ESM.pdf]

小脑认知情感综合征量表
(CCAS-SCALE)

姓名　　　　**年龄**
职业　　　　**受教育程度**
填表日期　　**性别**

表

项目	说明	原始分数	通过=0，失败=1
语意流畅性	说出26个名字是满分26分，≤15个为失败<br>（在右下角表示）<br>请在一分钟内尽可能多地说出动物或生物名字	/26	
语音流畅性	说出19个词语是满分19分，≤9个为失败<br>（在右下角表示）<br>请在一分钟内尽可能多地说出“发”开头的词语（人名、地名除外）	/19	
类别转换	说出15个交替是满分15分，重复或错误不计分，≤9个交替为错误（在右下角表示）<br>先说出一种蔬菜，然后说出一种职业或工作，再说出一种蔬菜，然后说出一种职业或工作，以此类推。请在一分钟内尽可能多地说出这种蔬菜与职业之间的转换。	/15	
口头词汇登记	这项测试不计分（进行4轮单词学习，确定大脑是否参与）<br>我将会读一组词语，然后由您重复这些词语。几分钟后我将会让你回忆这些词语。 （1秒读一个词语，患者重复一遍，然后再重复一遍，一直重复到患者想起这5个词语，第4轮尝试后停止）		
正向数字广度	分数为正确重复的数字串的数字数量，≤5个为失败<br>我将会读一串数字，请跟随我按顺序重复这串数字。（1秒读一个数字，从带星号的数字串开始。若受试者不能重复，则从带星号的数字串之前的数字串开始）<br>5-9[ ]　4-8-7-0*[ ]　3-0-1-2-6-4[ ]　2-0-5-6-9-7-3-8[ ]<br>2-1-3[ ]　1-6-9-2-5[ ]　7-3-1-9-8-4-6[ ]	/8	
逆向数字广度	分数为正确重复的数字串的数字数量，≤3个为失败，不能完成2个数字串倒数为0分。<br>请倒数这些数字。（给受试者举例子，然后从*开始）<br>（例：5-8=8-5）*6-1[ ]　3-8-2[ ]　4-7-0-9[ ]　6-5-2-8-1[ ]　5-9-0-3-7-4[ ]	/6	
画立方体	如果画出12条线，符合三维的图形，为15分。如果不是12条线或不符合三维的图形，则让受试者接下来复制图形。<br>请画一个立方体——一个六面盒子，画成透明的（画于左下角空白处）	/15	
复制立方体	12条线，每条线1分，不是三维图形减1分，少一条线减1分，多一条线减一分。≤11分为失败。<br>请依照下页立方体完全复制。（整洁度不计分）		

口头词汇登记：

	[鲜花]		[张建国]		[勇气]		[演讲]		[黄色]
第一轮	[ ]	-	[ ]	-	[ ]	-	[ ]	-	[ ]
第二轮	[ ]	-	[ ]	-	[ ]	-	[ ]	-	[ ]
第三轮	[ ]	-	[ ]	-	[ ]	-	[ ]	-	[ ]
第四轮	[ ]	-	[ ]	-	[ ]	-	[ ]	-	[ ]

画立方体

标记

语义流畅性	语音流畅性	类别转换

Hoche, Guell, Vangel, Sherman, Schmahmann
中文版翻译：南善姬, Jeremy D. Schmahmann
美国麻省总医院神经内科神经解剖学与小脑神经生物学实验室认知行为神经病学组共济失调中心
©2016 美国麻省总医院保留所有版权。

延迟回忆	自发回忆每个词语3分，类型提示回忆每个词语2分，多项选择提示回忆每个单词1分。总分为所有回忆单词分数相加。≤10分计为失败。如果多项选择提示下不能回忆2个以上单词，需注意大脑受累。	原始分数	通过=0，失败=1

请回忆之前让您复述的词语。（必要时可按左下角选择提示）

	[鲜花]	[张建国]	[勇气]	[演讲]	[黄色]
自发回忆	[ ]	[ ]	[ ]	[ ]	[ ]
类型提示回忆	[ ]	[ ]	[ ]	[ ]	[ ]
多项选择回忆	[ ]	[ ]	[ ]	[ ]	[ ]

/15

相似性 正确答案（概念）=2分，部分正确答案（具体细节）=1分，错误或无答案=0分，满分=8分，≤6分计为失败。参考答案在右下角。

以下词语有什么相似的地方？（先举例后测试）

（例：球/月球=圆形） 1、鼻子/耳朵 2、绵羊/大象 3、湖泊/河流 4、飞机/汽车

[_/2] [_/2] [_/2] [_/2]

/8

手指叩击 无错误2分，1个错误1分，2个或2个以上错误0分。0分计为失败。

当我敲1次时，请抬起你的手指，然后收回去。 当我敲2次时，什么都不用做。

(向受试者示范，确认其完全理解)

1-1-1-2-2-1-2-2-2-1-2-1-2-1

/2

情感 一个都没有为6分，有一个即减去1分，≤4分计为失败。

评分者评估是否存在以下情况。病人和/或照顾者共同完成评估。

[ ]注意力不集中或头脑不灵活
[ ]情绪不稳定，不协调的或不恰当的情绪，绝望或抑郁
[ ]容易出现感觉信息超载或避免与他人接触
[ ]表达出不和逻辑的想法或妄想
[ ]缺乏同情心，淡漠、情感迟钝
[ ]易怒，易激惹，逆反，识别社交线索和社会边界困难

/6

总分 /120 /10

计算第一栏的总分和第二栏的失败数量。
1项失败=可能CCAS; 2项失败=很可能CCAS; 3项或以上失败=肯定CCAS

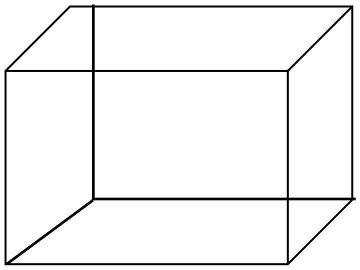

复制立方体图形

词语回忆测试的提示和选择项目					
测试单词	鲜花	王建国	勇气	演讲	黄色
线索提示	在花园里生长	男性的名字	特点或美德	交谈的方式	颜色
多项提示	树	李小明	勇敢	演讲	红色
	灌木丛	张国华	勇气	说话	绿色
	鲜花	赵长海	诚实	唱歌	蓝色
	草坪	王建国	耐心	喊叫	黄色

相似性	部分正确的概念回答
鼻子/耳朵	脸颊，身体的一部分
绵羊/大象	腿，尾巴
湖泊/河流	湿，冷，游泳
飞机/汽车	使用燃料，可以搭乘
