# Supplementary material for: Reliability and validity study of the Chinese version of the Cerebellar Cognitive Affective Syndrome Scale in patients with cerebellar injury
Source: Acta Neurol Belg. 2024 Jul 2;124(6):1867–73. doi: 10.1007/s13760-024-02594-x (PMC11615023; doi:10.1007/s13760-024-02594-x)
Supplement: Supplementary file 2 — Supplementary file2 (PDF 257 KB) [file 13760_2024_2594_MOESM2_ESM.pdf]

CEREBELLAR COGNITIVE AFFECTIVE / SCHMAHMANN SYNDROME SCALE (CCAS-Scale) VERSION 1A.

NAME:
ID#
DATE

DOB:
Education (Yrs)

Test	Instructions	RAW SCORE	PASS=0 FAIL=1
**SEMANTIC FLUENCY**	Score = total correct words (up to a maximum of 26 words). Fail if Score 15 or less. *(Use space bottom right for notation).*<br>Please name as many animals or living creatures as you can in one minute	/26	
**PHONEMIC FLUENCY**	Score = total correct words (up to a maximum of 19 words). Fail if Score 9 or less. *(Use space bottom right for notation).*<br>Please name as many words as you can in one minute that start with the letter F. Do not use names of people or places or repeat the same word in different forms.	/19	
**CATEGORY SWITCHING**	Score = total number of correct alternating words (up to a maximum of 15 alternations). Repetitions or set loss errors are not scored. Fail if Score 9 or less. *(Use space bottom right for notation).*<br>Please name a type of vegetable and then a type of profession or job, and then another vegetable and another profession, and so on, switching between the two lists. Name as many as you can in one minute.	/15	
**VERBAL REGISTRATION**	This test is not scored. (The need for 4 attempts to learn 5 words raises concern for cerebral involvement).<br>I am going to read you a list of words which I would like you to learn. Please repeat these words. I am going to ask you to give them back in a few minutes. *(Read 5 words at rate of 1 / second. Subject repeats them once, then repeats them again. Repeat trials until subject recalls all 5 words. Stop after 4 attempts.)*		

	[Flower]		[Robert]		[Courage]		[Speak]		[Yellow]
1st attempt	[ ]	-	[ ]	-	[ ]	-	[ ]	-	[ ]
2nd attempt	[ ]	-	[ ]	-	[ ]	-	[ ]	-	[ ]
3rd attempt	[ ]	-	[ ]	-	[ ]	-	[ ]	-	[ ]
4th attempt	[ ]	-	[ ]	-	[ ]	-	[ ]	-	[ ]

Test	Instructions	RAW SCORE	PASS=0 FAIL=1
**DIGIT SPAN FORWARD**	Score = maximum string of numbers correctly repeated. Fail if Score 5 or less.<br>I am going to read you some numbers. Please repeat them in exactly the same order *(Read aloud at a rate of 1 per second. Start with * and administer previous items if subject fails to repeat *).*<br>5-9 [ ] 4-8-7-0 * [ ] 3-0-1-2-6-4 [ ] 2-0-5-6-9-7-3-8 [ ]<br>2-1-3 [ ] 1-6-9-2-5 [ ] 7-3-1-9-8-4-6 [ ]	/8	
**DIGIT SPAN BACKWARD**	Score = maximum string of numbers correctly repeated. Fail if Score 3 or less. Inability to reverse 2 digits scores 0.<br>Now please say these numbers backwards, in reverse order. *(Give example, then start with *).*<br>(e.g., 5-8 = 8-5) *6-1 [ ] 3-8-2 [ ] 4-7-0-9 [ ] 6-5-2-8-1 [ ] 5-9-0-3-7-4 [ ]	/6	
**CUBE (DRAW)**	Score = 15 points if 12 lines present and diagram is 3-dimensional. If 12 lines not present or the diagram is not 3 dimensional, administer "CUBE (COPY)".<br>Please draw a cube – a six-sided box, make it transparent or see-through. *(Use space bottom left).*	/15	
**CUBE (COPY)**	Score = 12 points, 1 for each line. Deduct 1 point if not 3-D, 1 point for each line not drawn, 1 point for each additional line >12. Fail if Score 11 or less.<br>Please copy the cube shown on PAGE 2. *(Neatness not scored).*		

Draw cube here.

Notation:

Semantic Fluency	Phonemic Fluency	Category switching

		RAW SCORE	PASS=0 FAIL=1
**VERBAL RECALL**	Spontaneous = 3 points per word, category = 2 points , multiple choice = 1 point. Score = total points. Fail if Score 10 or less. Inability to recall more than 1 word from multiple choice raises concern for cerebral involvement.		
	What were the words I asked you to learn earlier? *(Subject recalls the words learned previously. Use cues and multiple choice alternatives bottom left if needed).*	/15	
**SIMILARITIES**	Correct answer (conceptual) = 2 points, partial answer (concrete) = 1 point, incorrect answer / no answer = 0 points. Score = total points. Fail if Score 6 or less. Key-bottom right.		
	How are the following words alike; what is the same about them? (*Provide example, then test items).*	/8	
**GO NO-GO**	2 points for no errors, 1 point for one error, 0 points for two or more errors. Score = total points. Fail if Score 0.		
	I am going to tap the table. When I tap once, please raise your finger then put it back down again. When I tap twice, don't do anything. *(Give an example of each condition to make sure subject understands).* 1 – 1 – 1 – 2 – 2 – 1 – 2 – 2 – 2 – 1 – 2 – 1 – 2 - 1	/2	
**AFFECT**	Score 6 points if none are present. Subtract 1 for each item present. Fail if Score 4 or less. *(Rater assesses if the following are present, incorporating input from patient and/or caregiver)*	/6	
	**TOTAL SCORE**	/120	/10

Verbal recall:

	[Flower]		[Robert]		[Courage]		[Speak]		[Yellow]
Spontaneous recall:	[ ]	-	[ ]	-	[ ]	-	[ ]	-	[ ]
Recall with category cue:	[ ]	-	[ ]	-	[ ]	-	[ ]	-	[ ]
Recall with multiple choice:	[ ]	-	[ ]	-	[ ]	-	[ ]	-	[ ]

Similarities:

(e.g., Ball/Moon = Round) 1.Nose/Ear [__/2] 2. Sheep/Elephant [__/2] 3. Lake/River [__/2] 4. Airplane/Motorcycle [__/2]

Affect:

[ ] Difficulty with focusing attention or mental flexibility
[ ] Emotionally labile, incongruous emotions, appears hopeless or depressed
[ ] Shows easy sensory overload or avoidant behaviors
[ ] Expresses illogical thoughts or paranoia
[ ] Lacks empathy, is apathetic, or has blunted affect
[ ] Angry or aggressive, irritable, oppositional, difficulty with social cues and social boundaries

Calculate total raw score (1st column) and total number of failed tests (2nd column).
1 failed test = Possible CCAS; 2 failed tests = Probable CCAS; 3 or more failed tests = Definite CCAS

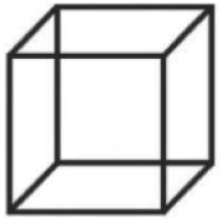

Copy the cube here.

CUES AND MULTIPLE CHOICE ITEMS FOR VERBAL RECALL TEST

Test word	Flower	Robert	Courage	Speak	Yellow
Cue	Grows in the garden	Boy's name	Trait or virtue	Way of communicating	Color
Multiple choice items	Tree	Stephen	Bravery	Speak	Red
	Bush	Michael	Courage	Talk	Green
	Flower	Joseph	Honesty	Sing	Blue
	Grass	Robert	Patience	Shout	Yellow

SIMILARITIES	Correct conceptual answers (examples)	Partial correct / concrete answers (examples)
Nose/Ear	Sense organs	Face, body part
Sheep/Elephant	Mammals, animals	Legs, tails
Lake/River	Bodies of water	Wet, cold, swim
Airplane/Motorcycle	Vehicles, transportation	Use fuel, ride them

Hoche, Guell, Vangel, Sherman, Schmahmann
Ataxia Unit, Cognitive Behavioral Neurology Unit, Schmahmann Laboratory for Neuroanatomy and Cerebellar Neurobiology, Department of Neurology, Massachusetts General Hospital. © 2017 The General Hospital Corporation. All Rights Reserved.
